# Favorable Response to Long-term Nucleos(t)ide Analogue Therapy in HBeAg-positive Patients with High Serum Fucosyl-Agalactosyl IgG

**DOI:** 10.1038/s41598-017-02158-5

**Published:** 2017-05-16

**Authors:** Cheng-Hsun Ho, Hung-Wen Tsai, Chen-Yeh Lee, Li-Juan Huang, Rong-Nan Chien, I-Chin Wu, Yen-Cheng Chiu, Wen-Chun Liu, Pin-Nan Cheng, Ting-Tsung Chang, Shu-Hui Chen

**Affiliations:** 1 Department of Internal Medicine, National Cheng Kung University Hospital, College of Medicine, National Cheng Kung University, Tainan, Taiwan; 2 Research Center of Clinical Medicine, National Cheng Kung University Hospital, College of Medicine, National Cheng Kung University, Tainan, Taiwan; 3 Department of Pathology, National Cheng Kung University Hospital, College of Medicine, National Cheng Kung University, Tainan, Taiwan; 4 Infectious Disease and Signaling Research Center, National Cheng Kung University, Tainan, Taiwan; 5 Department of Chemistry, National Cheng Kung University, Tainan, Taiwan; 6 Liver Research Unit, Chang Gung Memorial Hospital, College of Medicine, Chang Gung University, Keelung, Taiwan; 7 Institute of Molecular Medicine, College of Medicine, National Cheng Kung University, Tainan, Taiwan

## Abstract

Aberrant IgG glycosylation is a feature of hepatitis B virus (HBV) infection but its effect on a long-term efficacy of antiviral therapy has never been addressed. After a screening of 1,085 patients, 132 eligible HBV e antigen (HBeAg)-positive and 101 HBeAg-negative patients with anti-HBV nucleos(t)ide analogue monotherapy were enrolled with on-treatment follow-ups for at least one year. IgG1 *N*-glycome was profiled using mass spectrometry and evaluated for its relevance in treatment responses. The results indicated that a high level of serum fucosyl-agalactosyl IgG1 (IgG1-G0F) at baseline was associated with the severity of liver inflammation and damage but advanced treatment responses, including HBV DNA loss, HBeAg seroconversion, a reduced drug resistance rate, and a liver histological improvement at year 1, thereby improving the long-term treatment efficacy and the probability of treatment discontinuation in HBeAg-positive patients. Stepwise Cox regression analyses revealed that baseline IgG1-G0F >30% was an independent factor that links to virological response (HR 3.071, 95% CI 1.835–5.141, *P* < 0.001) or HBeAg seroconversion (HR 2.034, 95% CI 1.011–4.093, *P* = 0.046). Furthermore, a high IgG1-G0F level at the treatment endpoint was associated with an off-treatment sustained virological response. In conclusion, IgG1-G0F favors the medication outcome for HBeAg-positive chronic hepatitis B.

## Introduction

There are more than 300 million people worldwide have chronic hepatitis B (CHB) and over one million deaths from CHB-related liver cirrhosis or hepatocellular carcinoma every year^1, 2^. The current management of CHB involves an administration of nucleos(t)ide analogue (NA), which has advantages on a lower medical comorbidity and a better tolerance than interferon-based therapies. NA treatment suppresses hepatitis B virus (HBV) replication but not eliminates viruses, hence a long-term, even lifelong, usage of NA is necessary. Additionally, drug insusceptibility, antiviral resistance, and off-treatment virological relapse challenge an overall efficacy of NA therapy. The presence of favorable treatment responses, including undetectable HBV DNA in serum (virological response), HBV e antigen (HBeAg) seroconversion (development of antibodies against antigens), an effective decline or seroconversion of HBV surface antigen (HBsAg), and an improvement of liver injury, in patients with CHB rely on not only substantial suppression of virus replication but also host immune surveillance. It is well known that CHB patients who achieve rapid treatment response have a strikingly lower probability of disease progression and deterioration than those who have delayed or poor response^3, 4^. In contrast, patients who suffer an unsatisfactory therapeutic efficacy or a treatment failure are at a high risk of developing hepatitis flare, end-stage liver failure, or liver cancer^5, 6^. Given that the treatment response determines medication outcomes of CHB, it is imperative to identify a prognostic marker for early evaluating the efficacy of long-term NA therapy, thus optimizing the regimen.

Antibody-mediated immunity is vigorous in HBeAg-positive immune active phase of CHB^7^ and critical for the control of virus spreading. The pattern of *N*-linked glycosylation on the crystallizable fragment (Fc) of IgG (conserved at asparagine 297) determines the binding affinity of IgG to various Fcγ receptors (FcγRs) on immune cells and subsequently regulates downstream immune responses. Serum agalactosylated (G0) IgG, preferentially binding to activating FcγRs^8^, has a pro-inflammatory tendency and its increment is commonly linked to the progression of multiple autoimmune or infectious diseases^9–11^. We previously demonstrated an increment in serum agalactosylated IgG during CHB^12, 13^ and hypothesize that agalactosylated IgG may influence the efficacy of antiviral treatment owing to its immune modulation property. In this study, IgG bearing fucosyl-agalactosyl (IgG1-G0F) glycoform was identified to be strongly correlated with liver inflammation, which advantages virological, serological, and histological responses to long-term anti-HBV NA treatment in HBeAg-positive patients.

## Results

### Characteristics and serum IgG1 N-glycosylation profiles of patients

**Figure 1:**
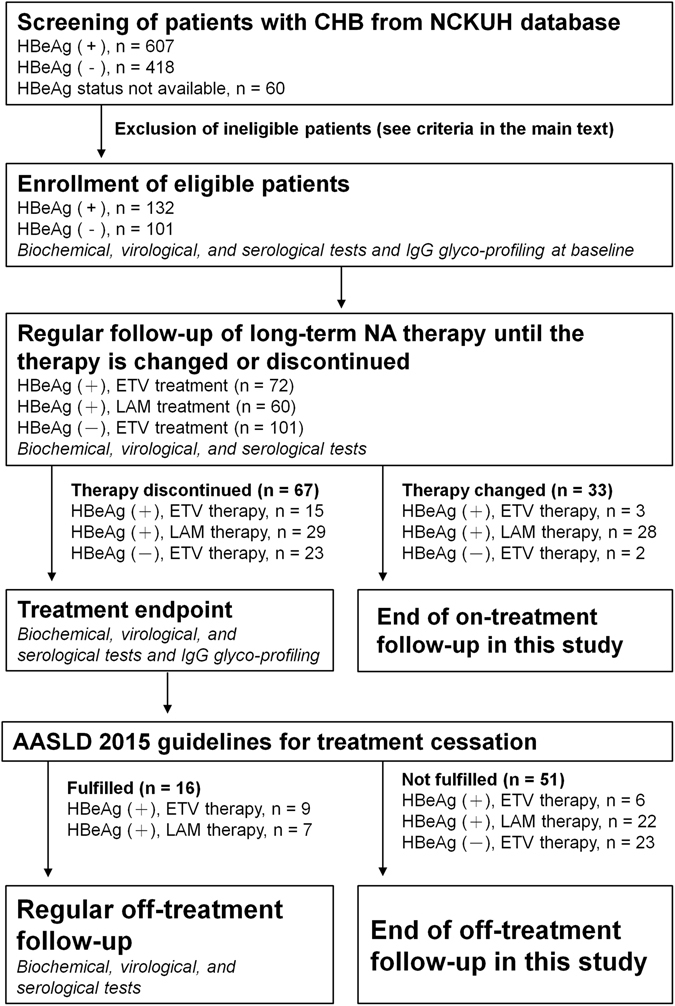
A flowchart of the enrollment, treatment, and follow-ups of patients.

A flowchart of this study was shown as Fig. 1. Clinical data of the patients are shown in Table 1. Two groups of HBeAg-positive patients had similar distributions of gender, age, and levels of alanine aminotransferase (ALT) and HBV DNA, at baseline. HBeAg-negative patients were older, as expected owing to the natural CHB history, than did the HBeAg-positive patients. Evaluations of treatment response at month 6 and year 1 revealed that entecavir was more potent than lamivudine on HBV DNA suppression but not on HBeAg seroconversion or HBsAg titer decline in HBeAg-positive patients. Moreover, the percentage of entecavir-related virological response was higher in HBeAg-negative patients than in HBeAg-positive patients. Primary treatment failure was detected in 10 HBeAg-positive patients with lamivudine treatment and one HBeAg-negative patient with entecavir treatment. Lamivudine resistance within the first year of treatment occurred in 14 HBeAg-positive patients. No entecavir-resistance cases were found.

**Table 1:** Characteristics of patients with chronic hepatitis B.

Variable	HBeAg-positive		HBeAg-negative	*P*-value 1	*P*-value 2
	ETV (n = 72)	LAM (n = 60)	ETV (n = 101)		
***Baseline***					
Sex (M:F)	47 : 25	38 : 22	73 : 28	0.856	0.403
Age (years)	46.8 ± 11.0	50.3 ± 9.8	53.1 ± 10.8	0.058	<0.001
ALT (U/L)	167.7 ± 142.1	134.5 ± 109.0	129.4 ± 112.1	0.141	0.049
AST (U/L)	79.0 ± 58.9	71.1 ± 50.4	82.3 ± 64.5	0.417	0.728
Albumin (g/dL)	4.4 ± 0.3	4.6 ± 0.3	4.2 ± 0.5	0.001	0.103
Total bilirubin (mg/dL)	0.8 ± 0.6	0.9 ± 0.3	1.0 ± 0.7	0.422	0.146
Total IgG (mg/mL)	11.6 ± 3.8	12.3 ± 3.2	11.7 ± 4.4	0.324	0.853
HBV DNA (Log_10_ IU/mL)	7.8 ± 1.7	8.2 ± 1.1	5.8 ± 1.5	0.071	<0.001
HBsAg (Log_10_ IU/mL)	4.0 ± 0.9	4.4 ± 0.7	3.2 ± 0.8	0.004	<0.001
On-treatment follow-up (years)	4.0 ± 2.0	4.4 ± 1.5	4.1 ± 1.8	0.134	0.707
Treatment discontinuation, n (%)	15 (20.8)	29 (48.3)	23 (22.8)	0.044	0.920
Post-treatment follow-up (years)	2.6 ± 1.9	3.0 ± 1.3	1.4 ± 1.0	0.451	0.041
***Evaluation of treatment response within 1 year***					
Normal ALT, n (%)					
6 months	47 (65.3)	41 (68.3)	64 (63.4)	0.711	1.000
1 year	58 (80.6)	36 (60.0)	81 (80.2)	0.022	0.849
HBV DNA decline (Δlog_10_ IU/ml)					
6 months	−5.2 ± 1.8	−3.7 ± 2.4	−4.3 ± 1.6	<0.001	0.001
1 year	−6.0 ± 1.9	−4.1 ± 2.7	−5.3 ± 1.7	<0.001	0.012
Virological response, n (%)					
6 months	17 (23.6)	5 (8.3)	43 (42.6)	0.021	0.010
1 year	36 (50.0)	11 (18.3)	81 (80.2)	<0.001	<0.001
HBeAg seroconversion, n (%)					
6 months	8 (11.1)	8 (13.3)	NA	0.791	NA
1 year	11 (15.3)	11 (18.3)	NA	0.648	NA
HBsAg decline (Δlog_10_ IU/mL)					
6 months	−0.4 ± 0.8	−0.4 ± 0.8	−0.2 ± 0.6	0.929	0.075
1 year	−0.6 ± 0.9	−0.5 ± 0.9	−0.3 ± 0.7	0.739	0.042
Primary treatment failure, n (%)	0 (0.0)	10 (16.7)	1 (1.0)	<0.001	1.000
Genotypic resistance, n (%)					
6 months	0 (0.0)	1 (1.7)	0 (0.0)	0.455	NA
1 year	0 (0.0)	14 (23.3)	0 (0.0)	<0.001	NA

Data are shown as ratio, percentage or mean ± standard deviation. *P*- values for comparisons of continuous variables and nominal variables are from 2-tailed Student’s *t* tests and Fisher’s exact tests, respectively. *P*-value 1 is the comparison in HBeAg-positive patients between entecavir treatment and lamivudine treatment. *P*-value 2 is the comparison of entecavir treatment between HBeAg-positive patients and HBeAg-negative patients. Abbreviations: ALT, alanine aminotransferase; AST, aspartate aminotransferase; ETV, entecavir; HBeAg, hepatitis B virus e antigen; HBsAg, hepatitis B virus surface antigen; HBV, hepatitis B virus; LAM, lamivudine; NA, not available. Virological response is defined as undetectable HBV DNA (<20 IU/ml) in serum. Primary treatment failure refers to the inability of the antiviral agent to reduce serum HBV DNA by ≧1 log_10_ IU/ml within the first six months of treatment.

**Figure 2:**
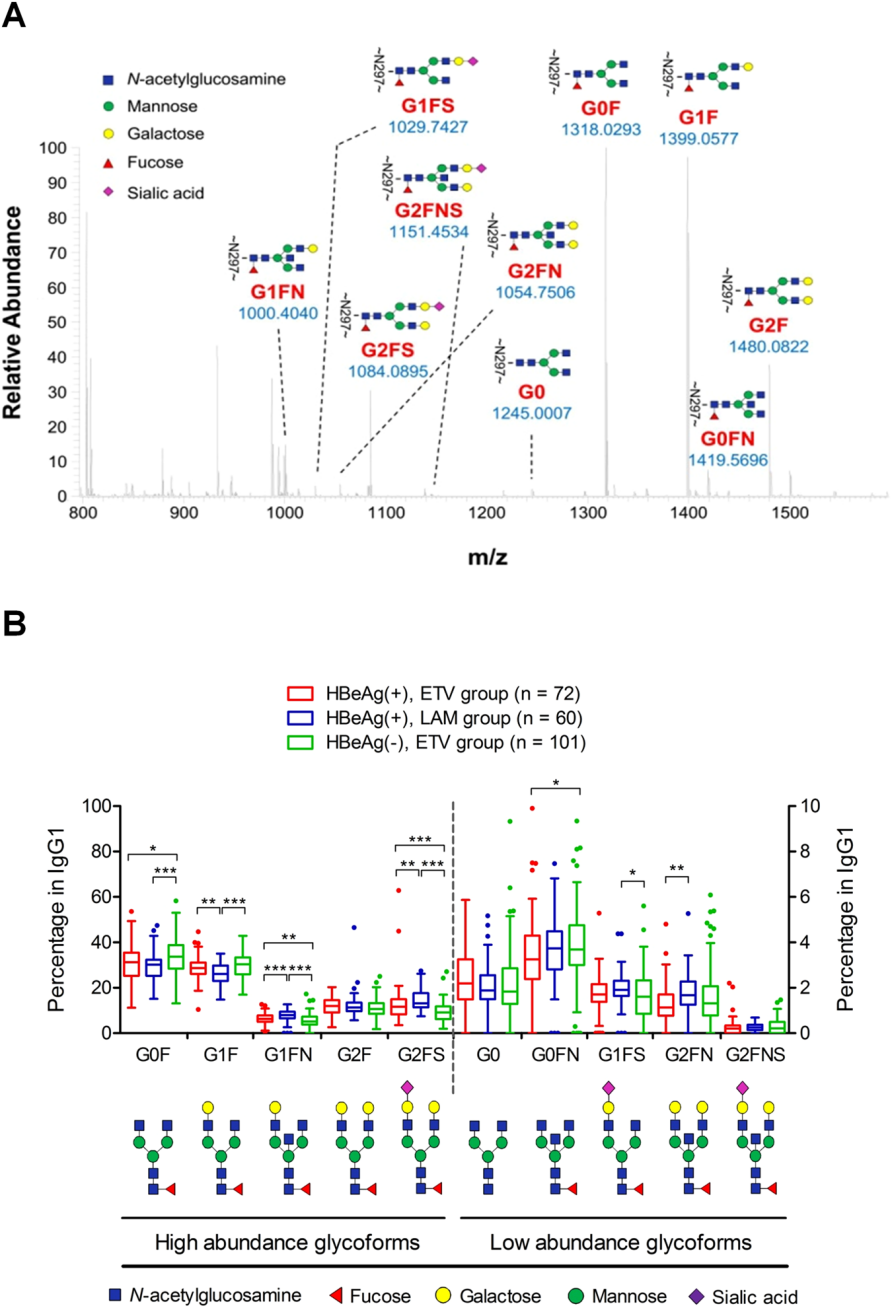
Serum IgG_1_ glyco-profiling by liquid chromatography–tandem mass in patients with chronic hepatitis B. (**A**) A representative mass spectrum of IgG1-Fc glycopeptide (EEQY**N**_**297**_STYR) with different *N*-glycoforms is shown. (**B**) Proportions of baseline serum IgG1-Fc glycoforms of patients are shown in Tukey box-and-whisker plots. *P*-values are obtained from Mann–Whitney *U* test (*P < 0.05; **P < 0.01; ***P < 0.001).

A representative MS1 spectrum that contains 10 major glycoforms on IgG1-Fc peptide backbone EEQY**N**_297_STYR is shown as Fig. 2A. The major glycan fragments detected using collision-induced dissociation-MS2 spectra confirmed the linkages of each glycoform (Supplementary Fig. 1). The IgG1 glyco-profiles in three groups of patients showed that the HBeAg-negative group had a higher level of G0F (fucosyl-agalactosyl) glycoform and lower levels of G1FN (fucosyl-bisected *N*-acetyl-glucosaminyl-partially galactosyl) and G2FS (fucosyl-sialyl-fully galactosyl) glycoforms than did the HBeAg-positive entecavir and lamivudine groups (Fig. 2B). Less heterogeneity of IgG1-Fc glyco-profiles was observed between 2 HBeAg-positive groups.

### IgG-G0F was correlated with liver inflammation and histological damage

**Figure 3:**
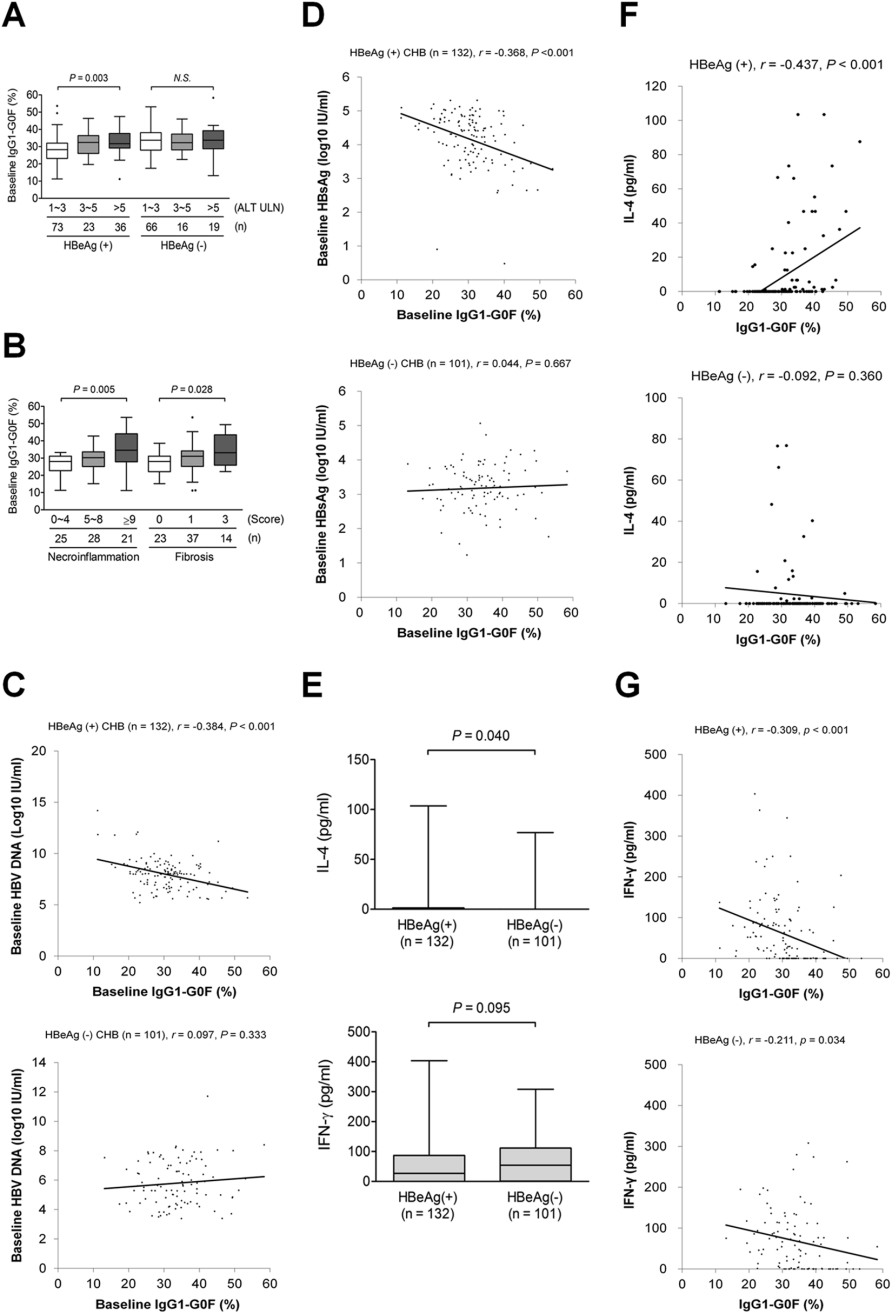
Correlation between serum IgG1-G0F and disease status during chronic hepatitis B. Baseline IgG1-G0F proportions in patients with different levels of (**A**) ALT or (**B**) liver histological damage are shown in Tukey box-and-whisker plots. Relationships between proportions of IgG1-G0F and levels of (**C**) HBV DNA or (**D**) HBsAg are shown. Expression levels of (**F**) IL-4 or IFN-γ, and correlations of IgG1-G0F with (**F**) IL-4 or (**G**) IFN-γ, in serum from HBeAg-positive patients and HBeAg-negative patients, are shown. *P*-values from (**A**,**B**) are obtained from Kruskal-Wallis tests. *P*-values from (**E**) are obtained from Mann–Whitney *U* tests. The coefficient *r* from (**C**,**D**,**F**,**G**) are taken from Pearson’s correlation tests. ALT, alanine aminotransferase; HBeAg, hepatitis B virus e antigen; HBeAg, hepatitis B virus surface antigen; IFN, interferon; IL, interleukin; NS, not significant; ULN, upper limits of normal.

At baseline, G0F was the only IgG1 glycoform that was positively correlated with levels of ALT (Fig. 3A) and liver necroinflammation/liver fibrosis (Fig. 3B), and was inversely correlated with HBV viral load (Fig. 3C) and HBsAg titer (Fig. 3D) in HBeAg-positive patients; nevertheless, these linkages were not seen in HBeAg-negative patients. Moreover, IgG1-G0F was not associated with age or sex in patients with CHB (Supplementary Fig. 2). Examinations of interleukin (IL)-4 and interferon (IFN)-γ, 2 essential activators for humoral immunity and CD8+ cytotoxic T cell, respectively, revealed that HBeAg-positive patients had a higher IL-4 level and a borderline lower IFN-γ level than HBeAg-negative patients (Fig. 3E). In HBeAg-positive patients, IgG1-G0F was positively correlated with IL-4 and negatively correlated with IFN-γ, respectively (Fig. 3F and G). Weak associations between IgG1-G0F and cytokines in HBeAg-negative patients were observed. These results indicated that an increase in IgG1-G0F level was linked to a severe liver necroinflammation but a lower HBV replication activity during the immune-active HBeAg-positive CHB.

### Effect of baseline IgG1-G0F on 1-year efficacy of NA treatment

**Figure 4:**
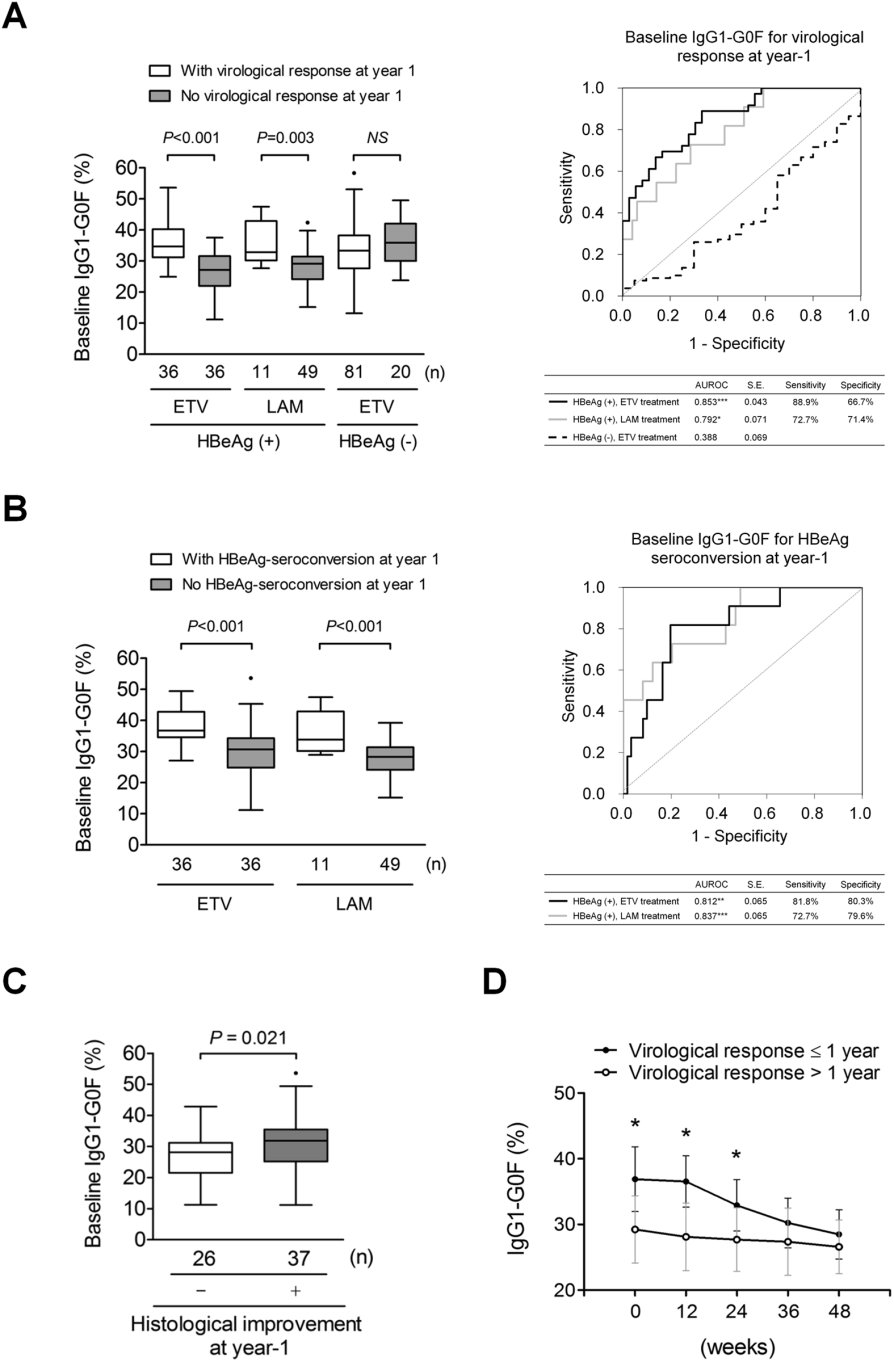
Evaluating 1 year of treatment efficacy using baseline IgG1-G0F. Proportions of baseline serum IgG1-G0F from patients with or without (**A**) virological response or (**B**) HBeAg seroconversion, and receiver operating characteristic (ROC) curves for baseline IgG1-G0F level to differentiate (**A**) virological response or (**B**) HBeAg seroconversion, after 1 year of treatment, are shown (*P < 0.05; **P < 0.01; ***P < 0.001). (**C**) Baseline IgG1-G0F proportions in HBeAg-positive patients with or without an improvement of liver injury at year 1 are shown in Tukey box-and-whisker plots. *P*-values are obtained from Mann–Whitney *U* tests. (**D**) Kinetics of serum IgG1-G0F from baseline to week 48 in HBeAg-positive patients with (n = 17) or without (n = 27) virological response within 1 year are shown in line chart with standard deviation. *P*-values are from Student’s *t* tests. ETV, entecavir; HBeAg, hepatitis B virus e antigen; LAM, lamivudine; NS, not significant; SE, standard error.

We quested whether IgG1-G0F had an effect on anti-HBV treatment. Logistic regression analyses revealed that G0F was an essential IgG1-Fc glycoform linking to virological response (undetectable HBV DNA in serum) in HBeAg-positive but not HBeAg-negative CHB (Supplementary Table 1). Moreover, baseline IgG1-G0F level was higher in HBeAg-positive patients who achieved virological response after 1 year of treatment than their counterparts (Fig. 4A). The areas under receiver operating characteristic curves (AUROC) for baseline IgG1-G0F level to differentiate virological response at year 1 was 0.792 (*P* < 0.05) in the HBeAg-positive lamivudine group and 0.853 (*P* < 0.001) in the HBeAg-positive entecavir group. The prediction power of the same model for HBeAg-negative CHB was poor. Furthermore, an increase in serum IgG1-G0F level at baseline was associated with HBeAg seroconversion (Fig. 4B), a low incidence of lamivudine-related primary treatment failure or drug resistance (Supplementary Table 2), and an amelioration of liver injury (Supplementary Table 3 and Fig. 4C), in HBeAg-positive patients at year 1. A kinetic analysis revealed that HBeAg-positive patients who achieved virological response within year 1 had a higher serum IgG1-G0F level from baseline to week 24 than those achieved virological response later (Fig. 4D). The magnitude of IgG1-G0F decline in virological responders was conspicuously larger than that in non-responders. The levels of IgG1-G0F at week 48 between 2 groups were similar. These results demonstrate that a high baseline IgG1-G0F level favored a short-term treatment response in patients with HBeAg-positive CHB.

### Baseline IgG1-G0F differentiates long-term NA treatment outcomes

**Figure 5:**
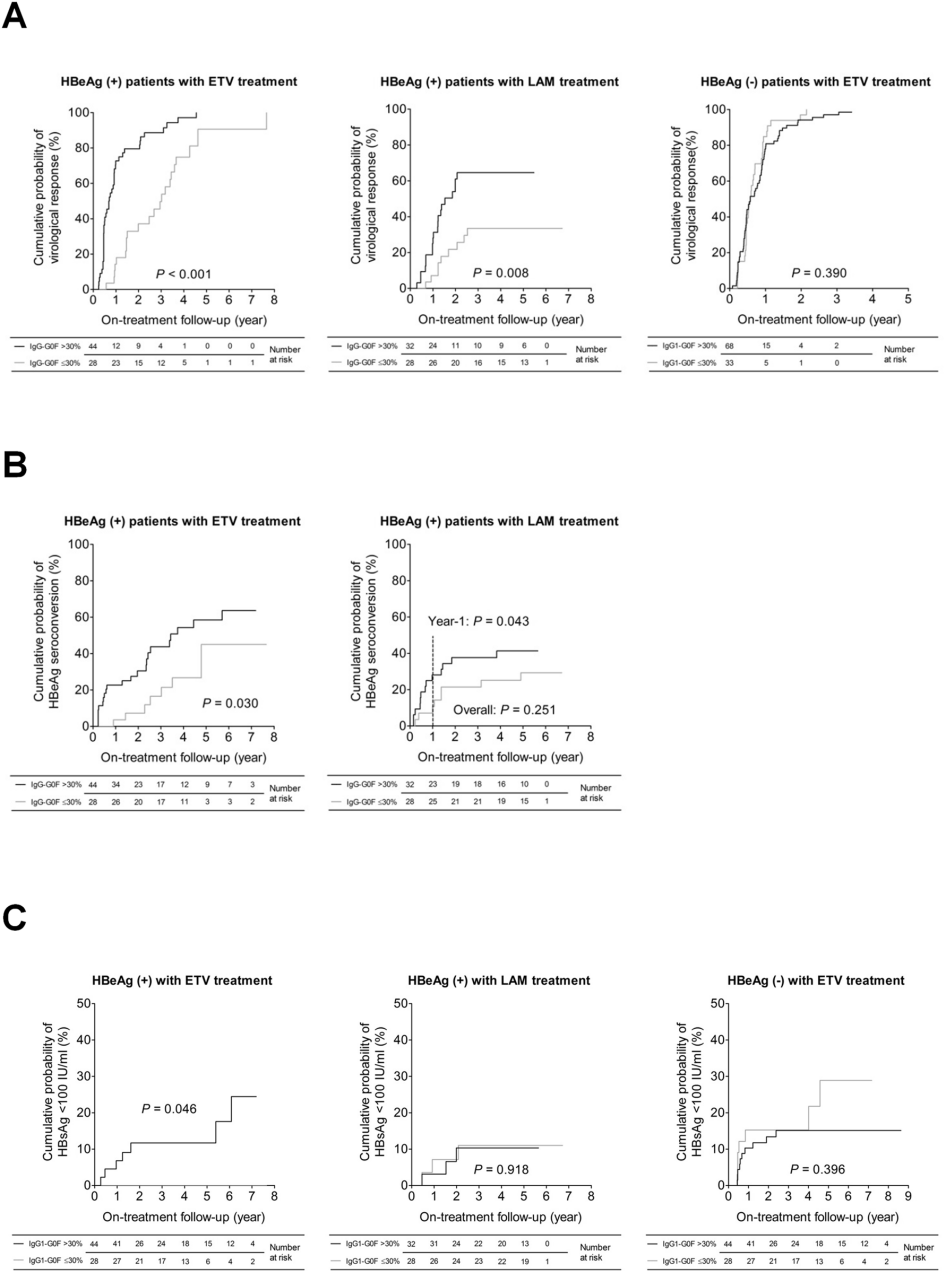
Baseline IgG1-G0F discriminates long-term efficacy of NA treatment in HBeAg-positive patients. Kaplan-Meier analyses of (**A**) virological response, (**B**) HBeAg seroconversion, and (**C**) HBsAg level <100 IU/ml after long-term treatment with and without baseline IgG1-G0F >30% are shown. *P*-values are obtained from log-rank tests. ETV, entecavir; HBeAg, hepatitis B virus e antigen; HBsAg, hepatitis B virus surface antigen; LAM, lamivudine.

A long-term follow-up revealed that entecavir was more potent on HBV suppression but had similar effects on HBeAg seroconversion, ALT normalization, and HBsAg reduction, when compared to lamivudine (Supplementary Fig. 3A–D). We set out to determine the impact of baseline IgG1-G0F on the long-term efficacy of NA treatment. Thirty percent is the median value of serum IgG1-G0F level at baseline in CHB patients and it was therefore set as a cut-off value of IgG1-G0F for following analyses. Results from Kaplan-Meier analyses showed that HBeAg-positive patients had a higher probability to achieve the virological response (log-rank *P* < 0.001 and *P* = 0.008 in the entecavir and lamivudine groups, respectively) when their baseline IgG1-G0F was greater than 30% (Fig. 5A). Again, this effect was not detected in HBeAg-negative CHB. The influence of IgG1-G0F on HBV suppression was not related to the gender of the patients (Supplementary Fig. 4). In HBeAg-positive patients, either male or female, IgG1-G0F exhibited its power to discriminate virological response during long-term treatment. A Cox regression analysis revealed that baseline IgG1-G0F >30% was an independent factor to differentiate virological response in HBeAg-positive CHB (Supplementary Table 4) and its effectiveness was even stronger when 32 cases with lamivudine resistance were excluded (Table 2).

**Table 2:** Cox regression analysis of favorable treatment response in HBeAg-positive patients without drug resistance (n = 100).

Variable	HBV DNA undetectable				HBeAg seroconversion			
	Univariate		Multivariate		Univariate		Multivariate	
	Hazard ratio	*P*-value	Hazard ratio	*P*-value	Hazard ratio	*P*-value	Hazard ratio	*P*-value
	(95% CI)		(95% CI)		(95% CI)		(95% CI)	
Sex (Male = 1. Female = 0)	1.184 (0.750–1.870)	0.469			1.404 (0.753–2.615)	0.286		
Age (years)	1.003 (0.982–1.024)	0.787			1.002 (0.974–1.030)	0.897		
ALT (U/L)	1.003 (1.001–1.004)	<0.001	1.002 (1.000–1.004)	0.016	1.002 (1.001–1.004)	0.005	1.002 (1.000–1.004)	0.041
HBV DNA (Log_10_ IU/mL)	0.753 (0.640–0.886)	<0.001	0.826 (0.675–1.011)	0.064	0.910 (0.740–1.120)	0.375		
HBsAg (Log_10_ IU/mL)	0.636 (0.524–0.773)	<0.001	0.776 (0.592–1.018)	0.067	0.729 (0.569–0.934)	0.012	0.733 (0.558–0.962)	0.025
Drug (ETV = 1, LAM = 0)	2.124 (1.233–3.659)	0.007	2.347 (1.321–4.168)	0.004	0.633 (0.342–1.171)	0.145		
Primary treatment failure* (Yes = 1, No = 0)	0.041 (0.001–1.771)	0.096			0.046 (0.000–20.959)	0.323		
IgG-G0F >30% (Yes = 1, No = 0)	3.358 (2.075–5.433)	<0.001	3.071 (1.835–5.141)	<0.001	2.823 (1.426–5.589)	0.003	2.034 (1.011–4.093)	0.046
HBeAg seroconversion (Yes = 1, No = 0)	2.369 (1.507–3.724)	<0.001	1.688 (1.024–2.783)	0.040				

Abbreviations: ALT, alanine aminotransferase; AST, aspartate aminotransferase; CI, confidence interval; ETV, entecavir; G0F, agalactosylation with core fucosylation; HBeAg, hepatitis B virus e antigen; HBsAg, hepatitis B virus surface antigen; HBV, hepatitis B virus; LAM, lamivudine. Primary treatment failure refers to the inability of the antiviral agent to reduce serum HBV DNA by ≧1 log_10_ IU/ml within the first six months of treatment. ^*^Only detected in the lamivudine group.

In addition to the virological response, an association of baseline IgG1-G0F >30% on HBeAg seroconversion was detected throughout the entire course of entecavir treatment but only within the first year of lamivudine treatment (Fig. 5B) owing to an accumulative lamivudine resistance with years (Supplementary Fig. 5A). After excluding the drug-resistant cases, IgG1-G0F regained its influence on HBeAg seroconversion during long-term lamivudine treatment (Supplementary Fig. 5B and Table 2). We next studied a relation between IgG1-G0F and HBsAg reduction. HBsAg seroconversion is an ideal treatment endpoint but is almost unattainable for CHB patients receiving NA treatment. In this study, only one HBeAg-positive patient with entecavir treatment was detected with HBsAg seroconversion. Accordingly, we examined whether baseline IgG1-G0F >30% was associated with a low HBsAg level (<100 IU/ml), a prelude of HBsAg loss and a prediction marker for lower risk of relapse^14–16^. Using Kaplan-Meier analyses, we found that baseline IgG1-G0F >30% increased the probability of HBsAg level <100 IU/ml in HBeAg-positive patients who received entecavir treatment (Fig. 5C). The data presented herein indicate that a high level of serum IgG1-G0F at baseline is correlated with favorable responses to long-term NA treatment, particularly entecavir, in HBeAg-positive patients.

### Association of IgG1-G0F with treatment discontinuation

**Figure 6:**
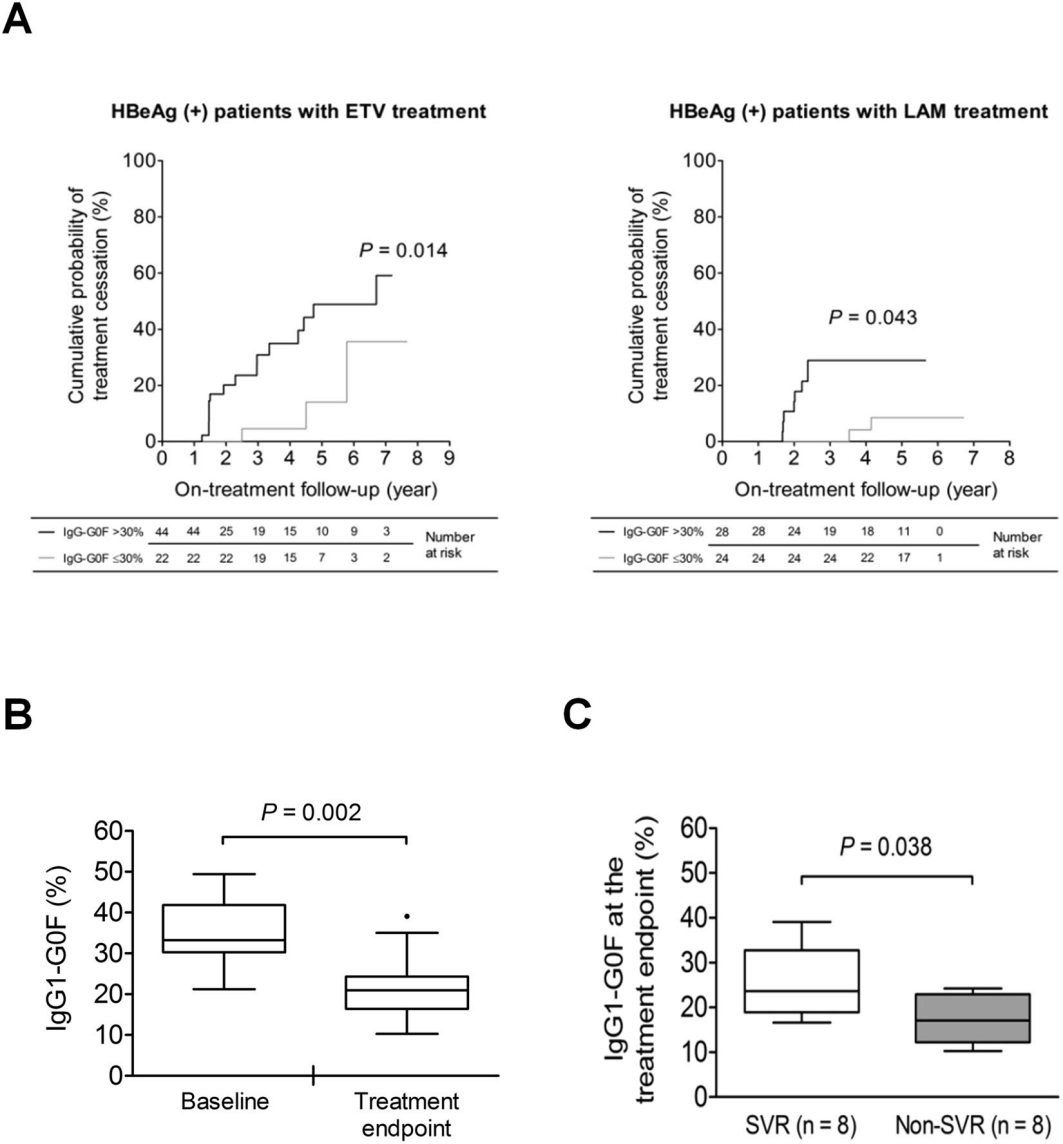
High baseline IgG1-G0F increases the probability of treatment discontinuation in HBeAg-positive patients. (**A**) Kaplan-Meier analyses of the probability of treatment discontinuation in HBeAg-positive patients with and without baseline IgG1-G0F >30% are shown. *P*-values are obtained from log-rank tests. Proportions of serum IgG1-G0F (**B**) between baseline and the treatment endpoint in 16 initially HBeAg-positive patients with treatment discontinuation or (**C**) at the treatment endpoint in HBeAg-positive patients with or without sustained virological response after treatment discontinuation, are shown as Tukey’s box-and-whisker plots. *P*-values in (**B**,**C**) are from Wilcoxon signed-rank test and Mann–Whitney *U* test, respectively. ETV, entecavir; HBeAg, hepatitis B virus e antigen; LAM, lamivudine; SVR, sustained virological response.

HBeAg-positive patients whose baseline IgG1-G0F >30% were exhibited to have a higher probability to achieve the treatment cessation criteria for CHB (log-rank *P* = 0.014 for entecavir treatment and *P* = 0.043 for lamivudine treatment) (Fig. 6A). We next assessed a post-treatment effect of IgG1-G0F in 16 initially HBeAg-positive patients (9 received entecavir treatment and 7 received lamivudine treatment) who conformed guidelines from American Association for the Study of Liver Diseases (AASLD)^17^ and terminated NA treatment after a mean of 3.6 years of treatment duration. Their serum IgG1-G0F levels decreased from a median value of 33.2% at baseline to 21.0% at the treatment endpoint (Fig. 6B). Interestingly, the level of IgG1-G0F at the treatment endpoint was higher (*P* = 0.038) in the patients who achieved a sustained virological response (SVR) than those without SVR (Fig. 6C).

## Discussion

Mass spectrometry is a powerful tool for discovering novel biomarkers, particularly those are arduous to be detected using immunoassays, for example, IgG-Fc *N*-glycome. Purification of intact IgG bearing specific glycoform and the development of related detection kits remain substantial challenges due to a steric hindrance (glycans were embedded between two Fc domains) and a low immunogenicity of Fc glycopeptides. Fortunately, the high abundance (normal range: 5–15 mg/ml) and long half-life (>20 days except IgG subclass 3) of IgG in serum and the prevalence of mass spectrometry in medical labs nowadays make it highly feasible to include serum IgG glycosylation as a routine clinical test.

We previous reported an enhancement of serum agalactosylated IgG in patients with HBV-related liver diseases when compared to healthy individuals^12, 13^ and herein identified that G0F, the major (>80%) agalactosylated glycoform of IgG, favored the efficacy of anti-HBV therapy for immune active CHB. The present paper, to our best knowledge, is the first longitudinal study that reports the relevance of specific IgG glycan structure on long-term treatment efficacy. It is a well-known fact that agalactosylated IgG is pro-inflammation-prone owing to its ability to trigger various events including mannan-binding lectin-mediated complement cascade, type I IFN secretion, and antibody-dependent cellular cytotoxicity, to name a few^18–22^. In the present study, the thriving of agalactosylated IgG was closely associated with an increase in ALT level and severe liver inflammation during the immune-active phase of CHB; however, inflammatory immune responses elicited by agalactosylated IgG, on the other hand, may enhance the control of HBV replication and eliminate virus-infected hepatocytes in addition to NA treatment, thereby facilitating a mitigation of liver injury. That’s why HBeAg-positive patients with a high baseline IgG1-G0F level had a high grade of liver necroinflammation or fibrosis before treatment but had better on-treatment virological, serological, and histological responses. The kinetics of IgG1-G0F, which showed that virological responders had an overall higher level but a striking decline in IgG1-G0F than non-responders, authentically reflects the disease status and treatment responses of HBeAg-positive CHB. An increasing number of testimonies have pointed out a double-edged effect of antibody-mediated immunity upon HBV infection^23–29^. Our previous report^30^ demonstrating that patients with CHB who had a higher baseline level of ALT responded stronger to entecavir treatment could also support this phenomenon. IgG1-G0F is unlikely to possess a direct antiviral activity but one may plausibly speculate that humoral immunity, driven by IgG1-G0F, advances HBV clearance. Of note, the change in serum IgG1-G0F level during CHB was subtle but it has a profound influence on medication outcomes, suggesting that the immune modulatory effect of IgG glycan is tremendous and it is not allowed to be greatly fluctuated.

The development of drug-resistant HBV mutations depends on a replication advantage of viruses by conquering the immune selection pressure. Our findings indicate that immune activities induced by abundant IgG1-G0F may not only improve HBV clearance but also restrain the early expansion of drug-resistance mutant clones. Nevertheless, HBV mutants that gain survival advantages may accumulate and they are no longer quelled by host immune system after years. Therefore, the prediction power of IgG1-G0F on long-term lamivudine treatment is comparatively low.

Interestingly, the influence of IgG1-G0F on NA treatment was not evidenced in HBeAg-negative patients. During HBeAg-negative CHB, CD8^+^ T cell-mediated cytotoxicity, rather than innate or humoral immunity, takes over the control against HBV^31^ and IgG1-G0F, in this circumstance, contributes less to immune activation.

Additionally, a lower baseline HBV DNA level and predominant entecavir usage of HBeAg-negative cases in this study made it much easier to achieve the virological response, thus concealing the effect of IgG1-G0F. IL-4 and IFN-γ are pivotal cytokines for activating B cell differentiation and CD8+ T cell cytotoxicity, respectively, and they are essential for controlling HBV infection^32, 33^. Our results regarding IL-4 activation and its relationship to IgG-G0F in HBeAg-positive patients fortify the significance of IgG-mediated humoral immunity, which is tightly regulated by Fc *N*-glycans, in HBeAg-positive CHB. These manifestations, in correspondence with a report by Vanwolleghem *et al*.^7^ in which dominant expressions of immunoglobulin and B-cell function-related genes were exhibited in the immune active phase of CHB. According to a 2011 report by Wang *et al*.^34^, IL-4 or IFN-γ, together with stimuli comprising B cell receptor-triggering, CD40 stimulation, IL-2, and IL-10, did not affect the galactose content of IgG that was yielded from primary human CD19^+^ B cells. However, it should be noted that *in vivo* connections between liver and B cells, as well as the balance between cellular and humoral immunities, are more complicated than a simple positive or negative regulatory loop that was concluded *in vitro*.

The high cost of the long-term, even lifelong, usage of NA is strikingly a financial burden for patients with CHB, particularly those from developing countries. The AASLD guidelines suggest that clinicians may consider stopping NA treatment in HBeAg-positive patients who have undergone HBeAg seroconversion with undetectable HBV DNA for at least 12 months or had HBsAg loss^17^. In this study, a high baseline level of IgG1-G0F was shown to promote an earlier achievement of treatment cessation threshold in HBeAg-positive patients because of its effectiveness on virological and serological responses. Furthermore, a high level of IgG1-G0F at the treatment endpoint was correlated with SVR in patients who discontinued NA treatment, delineating a drug-independent manner of IgG1-G0F against HBV infection. The enrollment of more patients with NA discontinuation is needed to study the effect of IgG1-G0F on off-treatment HBV relapse or hepatitis flare.

In conclusion, HBeAg-positive patients with a high level of IgG1-G0F had favorable on-treatment and off-treatment responses to long-term nucleos(t)ide analogue therapy. We believe that this clinical glycoproteomic study is of great importance to not only early predict the long-term treatment efficacy for CHB but also prospect the development of new anti-HBV therapeutics that target antibody glycome.

## Materials and Methods

### Study design and patients

This retrospective cohort study was approved by the Institutional Review Board of National Cheng Kung University Hospital (NCKUH) and conducted in accord with the guidelines of the Declaration of Helsinki. Eligible patients had well-preserved serum samples, HBV DNA (>20,000 IU/mL in HBeAg-positive patients and >2,000 IU/mL in HBeAg-negative patients), HBV surface antigen (HBsAg) for more than six months, and they were negative for hepatitis C virus, human immunodeficiency virus, alcoholism- or autoimmune-induced liver diseases, liver cirrhosis, biliary disorders, symptoms of tuberculosis, rheumatoid arthritis, juvenile onset chronic arthritis, systemic lupus erythematosus, and Crohn’s disease. A screening of 1,085 CHB patients from NCKUH database was performed and a total of 132 HBeAg-positive and 101 HBeAg-negative patients were finally selected, of whom 44 HBeAg-positive and 38 HBeAg-negative patients have been described previously^12, 13^. Informed consent was obtained from all subjects. Seventy-two and 60 HBeAg-positive patients received entecavir and lamivudine monotherapy, respectively, and all of the HBeAg-negative patients received entecavir monotherapy. A regular on-treatment follow-up for at least 48 weeks was performed in all patients until the therapy was changed or discontinued. An off-treatment follow-up was performed in 16 initially HBeAg-positive patients who conformed guidelines of treatment cessation from AASLD^17^.

### Serological and virological tests

Detections of serum HBV DNA, HBeAg, HBsAg, or IgG levels have been previously described^12^. Virological response is defined as undetectable HBV DNA (<20 IU/ml) in serum. HBeAg seroconversion refers to the loss of HBeAg and the presence of antibodies against HBeAg. Sustained virological response (SVR) is defined as undetectable HBV DNA in serum after 24 weeks of treatment discontinuation. Primary treatment failure refers to the inability of the treatment to reduce serum HBV DNA by ≧1 log_10_ IU/ml within the first 6 months. The presence of lamivudine resistance-associated HBV clones was verified by a gene mutation and a change in the deduced amino acid residue in the YMDD motif on the viral polymerase gene. Levels of interleukin-4 and interferon-γ in sera of patients were detected using Ready-Set-Go ELISA kits (eBioscience, San Diego, CA).

### Liver Histology

Liver biopsy was carried out on 74 HBeAg-positive patients at baseline and 63 of them after 1 year of treatment. Liver histological damage was evaluated according to Knodell histology activity index (HAI), by a single experienced hepatopathologist who was blinded to the treatment assignment, biopsy sequence, and clinical outcome. A liver histological improvement was characterized as a decrease in the fibrosis score or in total necroinflammation score ≧2 without exacerbation of fibrosis.

### Serum IgG1-Fc glyco-profiling

Investigators were blinded to any clinical information of the patients when analyzing IgG glyco-profiles. In-gel trypsinized serum IgG heavy chain was injected into a high performance liquid chromatography (Model 1200; Agilent, Santa Clara, CA) which was equipped with a nano column (75 μm i.d. × 25 cm, 1.7 μm BEH130 C18, Waters Corporation, Milford, MA) and coupled online with an LTQ-Orbitrap XL mass spectrometer (Thermo Fisher Scientific, San Jose, CA). Mobile phase A was 0.1% fluoroacetic acid and mobile phase B was 0.1% fluoroacetic acid in acetonitrile. The gradient consisted of (1) 5 minutes at 2% B for sample loading in a pre-column, (2) increasing linearly from 2% to 40% B over 40 minutes, (3) increasing linearly from 40% to 80% B over 10 minutes, and finally (4) isocratic elution at 80% B for 10 minutes. The flow rate of the pre-column was 1 μL/minute for sample loading, and the nano-column was maintained at 300 nL/minute for separation. The retention time was recorded when the sample was injected. The LTQ-Orbitrap XL MS was operated in a data-dependent mode as follows: survey full-scan MS spectra (*m/z* 300–2000) were acquired in the Orbitrap with a mass resolution of 60,000 at *m/z* 400 (with an ion target value of 5 × 10^5^ ions), which was followed by five sequential collision-induced dissociation-MS^2^ using 35% of normalized collision energy in LTQ. The spectra that were generated in the collision-induced dissociation-MS^2^ step were searched using Mascot program (version 2.3, Matrix Science Ltd., London, UK) against the SwissProt 20110921 (532,146 sequences; 188,719,038 residues) protein databank for Homo sapiens using a mass tolerance of ±10 ppm for precursor ions and ±0.8 Da of product ions; significance was set at *P* < 0.05 for the initial filtering. Selected ion chromatograms of different glycoforms attaching to the identified peptide backbone (EEQY**N**_297_STYR) were extracted from the raw data. The abundance of a particular glycoform on EEQY**N**_297_STYR backbone was estimated from the peak height or peak area divided by the sum of peak height/area of all major glycoforms extracted from the chromatogram acquired from the same run. The percentage of each glycoform was obtained from an average of three LC-MS runs. MS^2^ spectra of each extracted glycopeptide were manually inspected to match all highly abundant product ions with a precursor ion mass accuracy <5 ppm to confirm their assignments. Ten major serum IgG-Fc glycoforms were analyzed in all participants. Other very low-abundant IgG-Fc glycoforms, such as those with tri-antennary, tetra-antennary, and high mannosylation, were preliminarily excluded.

### Statistical analysis

SPSS 17.0 for Windows was used for all statistical analyses. Continuous variables were compared using Student’s *t* tests or Mann–Whitney *U* tests for two independent groups and Kruskal-Wallis tests for three groups. Nominal variables were compared using Fisher’s exact tests or Pearson Chi-square tests. The Pearson correlation coefficient (*r*) was used to evaluate the relationship between two groups. A receiver operator characteristic (ROC) curve was plotted for baseline serum IgG1-G0F to differentiate virological response or HBeAg seroconversion in CHB patients after 1 year of NA treatment. A multivariate logistic regression analysis was performed to evaluate factors that were associated with lamivudine-related primary treatment failure or 1-year drug resistance in HBeAg-positive patients. Stepwise multivariate Cox regression analyses were performed to evaluate factors that were associated with virological response and HBeAg seroconversion after long-term NA treatment. Kaplan-Meier analyses and log-rank tests were used to assess the significance of IgG1-G0F on treatment responses. Significance was defined as *P* < 0.05, and all *P*-values were two-tailed.

## Electronic supplementary material

Supplementary file
