# Supplementary material for: Favorable Response to Long-term Nucleos(t)ide Analogue Therapy in HBeAg-positive Patients with High Serum Fucosyl-Agalactosyl IgG
Source: Sci Rep. 2017 May 16;7:1957. doi: 10.1038/s41598-017-02158-5 (PMC5434008; doi:10.1038/s41598-017-02158-5)
Supplement: Supplementary file 1 — Supplementary file [file 41598_2017_2158_MOESM1_ESM.pdf]

Supplementary File

Favorable Response to Long-term Nucleos(t)ide Analogue Therapy in HBeAg-positive Patients with High Serum Fucosyl-Agalactosyl IgG

Cheng-Hsun Ho[1,2], Hung-Wen Tsai[3,4], Chen-Yeh Lee[5], Li-Juan Huang[5], Rong-Nan Chien[6], I-Chin Wu[1,4], Yen-Cheng Chiu[1], Wen-Chun Liu[1,4], Pin-Nan Cheng[1], Ting-Tsung Chang[1,4,7,‡], and Shu-Hui Chen[5,‡,*]

[1]Department of Internal Medicine, National Cheng Kung University Hospital, College of Medicine, National Cheng Kung University, Tainan, Taiwan

[2]Research Center of Clinical Medicine, National Cheng Kung University Hospital, College of Medicine, National Cheng Kung University, Tainan, Taiwan

[3]Department of Pathology, National Cheng Kung University Hospital, College of Medicine, National Cheng Kung University, Tainan, Taiwan

[4]Infectious Disease and Signaling Research Center, National Cheng Kung University, Tainan, Taiwan

[5]Department of Chemistry, National Cheng Kung University, Tainan, Taiwan

[6]Liver Research Unit, Chang Gung Memorial Hospital, College of Medicine, Chang

Gung University, Keelung, Taiwan

[7]Institute of Molecular Medicine, College of Medicine, National Cheng Kung University, Tainan, Taiwan

Email: shchen@mail.ncku.edu.tw

‡ These authors contributed equally.

Supplementary Table 1. Logistic regression analysis of serum IgG glycoforms for virological response

	HBeAg-positive, ETV treatment (n = 72)		HBeAg-positive, LAM treatment (n = 60)		HBeAg-negative, ETV treatment (n = 101)	
IgG-glycoform	Odds ratio (95% CI)	*P*-value	Odds ratio (95% CI)	*P*-value	Odds ratio (95% CI)	*P*-value
Univariate						
**G0F**	**1.141 (1.017 - 1.280)**	**0.025**	**1.270 (1.101 - 1.464)**	**< 0.001**	0.997 (0.773 - 1.234)	0.844
G1F	1.009 (0.866 - 1.175)	0.911	0.962 (0.850 - 1.090)	0.546	0.970 (0.652 - 1.444)	0.881
G1FN	1.082 (0.777 - 1.505)	0.642	0.932 (0.772 - 1.126)	0.465	0.852 (0.513 - 1.414)	0.535
G2F	0.866 (0.700 - 1.072)	0.186	**0.806 (0.664 - 0.979)**	**0.030**	1.283 (0.682 - 2.416)	0.440
G2FS	0.952 (0.891 - 1.016)	0.139	0.918 (0.820 - 1.029)	0.141	1.229 (0.646 - 2.338)	0.530
G0	0.993 (0.538 - 1.836)	0.983	0.823 (0.498 - 1.362)	0.449	0.655 (0.297 - 1.443)	0.293
G0FN	1.243 (0.769 - 2.010)	0.374	1.020 (0.721 - 1.442)	0.913	0.931 (0.444 - 1.953)	0.850
G1FS	0.528 (0.232 - 1.202)	0.128	0.868 (0.447 - 1.684)	0.676	1.373 (0.170 - 11.091)	0.766
G2FN	0.492 (0.226 - 1.070)	0.074	0.617 (0.338 - 1.124)	0.115	1.084 (0.215 - 5.472)	0.922
G2FNS	0.868 (0.120 - 6.262)	0.889	0.229 (0.010 - 5.383)	0.360	1.890 (0.002 - 2000.74)	0.858
Multivariate						
**G0F**			**1.255 (1.078 - 1.462)**	**0.003**		
G2F			0.959 (0.783 - 1.176)	0.688		

Abbreviations: F, fucose; G0, agalactosylation; G1, partial galactosylation; G2, full galactosylation; N, *N*-acetylglucosamine; S, sialic acid.

Supplementary Table 2. Multivariate logistic regression analysis of primary treatment failure or one-year drug resistance in HBeAg-positive patients with lamivudine treatment (n = 60)

Variable	Primary treatment failure		1-year drug resistance	
	Odds ratio (95% CI)	*P*-value	Odds ratio (95% CI)	*P*-value
Sex (Male = 1. Female = 0)	5.188 (0.720 - 37.385)	0.102	2.296E9 (0.000 -)	0.998
Age (years)	0.923 (0.844 - 1.011)	0.085	1.018 (0.945 - 1.097)	0.634
ALT (U/L)	1.000 (0.991 - 1.009)	0.931	0.999 (0.990 - 1.008)	0.856
HBV DNA (Log_{10} IU/mL)	0.589 (0.186 - 1.865)	0.368	0.455 (0.157 - 1.317)	0.146
HBsAg (Log_{10} IU/mL)	1.957 (0.286 - 13.393)	0.494	1.558 (0.270 - 8.994)	0.620
IgG-G0F (%)	0.793 (0.657 - 0.957)	0.016	0.847 (0.721 - 0.994)	0.042

Primary treatment failure refers to the inability of the antiviral agent to reduce serum HBV DNA by $\geqq$1 log_{10} IU/ml within the first six months of treatment.

Abbreviations: ALT, alanine aminotransfease; AST, aspartate aminotransferase; CI, confidence interval; G0F, agalactosylation with core fucosylation; HBeAg, hepatitis B virus e antigen; HBsAg, hepatitis B virus surface antigen; HBV, hepatitis B virus.

Supplementary Table 3. Liver histological data from Knodell histology activity index in patients with HBeAg-positive chronic hepatitis B

Variable	Baseline (n = 74)	Year 1 (n = 63)	*P*-value
Periportal ± bridging Necrosis (0 : 1 : 3 : 4 : 5 : 6), n	30 : 17 : 13 : 6 : 4 : 4	43 : 12 : 7 : 1 : 0 : 0	0.002
Intralobular degeneration and focal necrosis (0 : 1 : 3 : 4), n	3 : 44 : 17 : 10	5 : 44 : 13 : 1	0.056
Portal inflammation (0 : 1 : 3 : 4), n	3 : 19 : 29 : 23	10 : 32 : 19 : 2	< 0.001
Fibrosis (0 : 1 : 3 : 4), n	23 : 37 : 14 : 0	15 : 38 : 10 : 0	0.685
Total score	7 (0 - 17)	3 (1 - 13)	< 0.001[a]

P-values for comparing individual factor between baseline and year-1 are obtained from Pearson Chi square tests.

[a]*P*-value for comparing the total score between baseline and year-1 in 63 patients is obtained from the Wilcoxon signed-rank test.

Abbreviation: HBeAg, hepatitis B virus e antigen.

Supplementary Table 4. Cox regression analysis of favorable treatment response in HBeAg-positive patients (n = 132)

	HBV DNA undetectable				HBeAg seroconversion			
	Univariate		Multivariate		Univariate		Multivariate	
Variable	Hazard ratio (95% CI)	*P*-value	Hazard ratio (95% CI)	*P*-value	Hazard ratio (95% CI)	*P*-value	Hazard ratio (95% CI)	*P*-value
Sex (Male = 1. Female = 0)	0.922 (0.606-1.403)	0.705			1.339 (0.739-2.429)	0.336		
Age (years)	1.000 (0.980-1.020)	0.973			0.989 (0.963-1.017)	0.441		
ALT (U/L)	1.003 (1.002-1.005)	< 0.001	1.002 (1.000-1.003)	0.043	1.003 (1.002-1.005)	< 0.001	1.002 (1.001-1.004)	0.010
HBV DNA (Log_{10} IU/mL)	0.708 (0.600-0.835)	< 0.001	0.812 (0.671-0.983)	0.032	0.822 (0.664-1.019)	0.074		
HBsAg (Log_{10} IU/mL)	0.583 (0.482-0.704)	< 0.001	0.802 (0.621-1.037)	0.092	0.642 (0.510-0.808)	< 0.001	0.681 (0.528-0.879)	0.003
Drug (ETV = 1, LAM = 0)	2.965 (1.894-4.639)	< 0.001	2.035 (1.152-3.595)	0.014	1.277 (0.723-2.255)	0.400		
Primary treatment failure* (Yes = 1, No = 0)	0.037 (0.003-0.490)	0.012	0.000 (0.000-1.3E301)	0.970	0.369 (0.089-1.524)	0.168		
Drug resistance* (Yes = 1, No = 0)	0.285 (0.155-0.525)	< 0.001	0.734 (0.324-1.661)	0.458	0.258 (0.102-0.653)	0.004	0.349 (0.133-0.911)	0.032
IgG-G0F > 30%	2.868	< 0.001	2.638	< 0.001	2.104	0.016	1.520	0.185

(Yes = 1, No = 0)	(1.847-4.454)		(1.639-4.247)		(1.148-3.858)		(0.818-2.825)	
HBeAg seroconversion	2.835	< 0.001	1.806	0.017				
(Yes = 1, No = 0)	(1.866-4.308)		(1.109-2.940)					

Abbreviations: ALT, alanine aminotransferase; AST, aspartate aminotransferase; CI, confidence interval; ETV, entecavir; G0F, agalactosylation with core fucosylation; HBeAg, hepatitis B virus e antigen; HBsAg, hepatitis B virus surface antigen; HBV, hepatitis B virus; LAM, lamivudine. Primary treatment failure refers to the inability of the antiviral agent to reduce serum HBV DNA by ≧1 $\log_{10}$ IU/ml within the first six months of treatment. *Only detected in the lamivudine group.

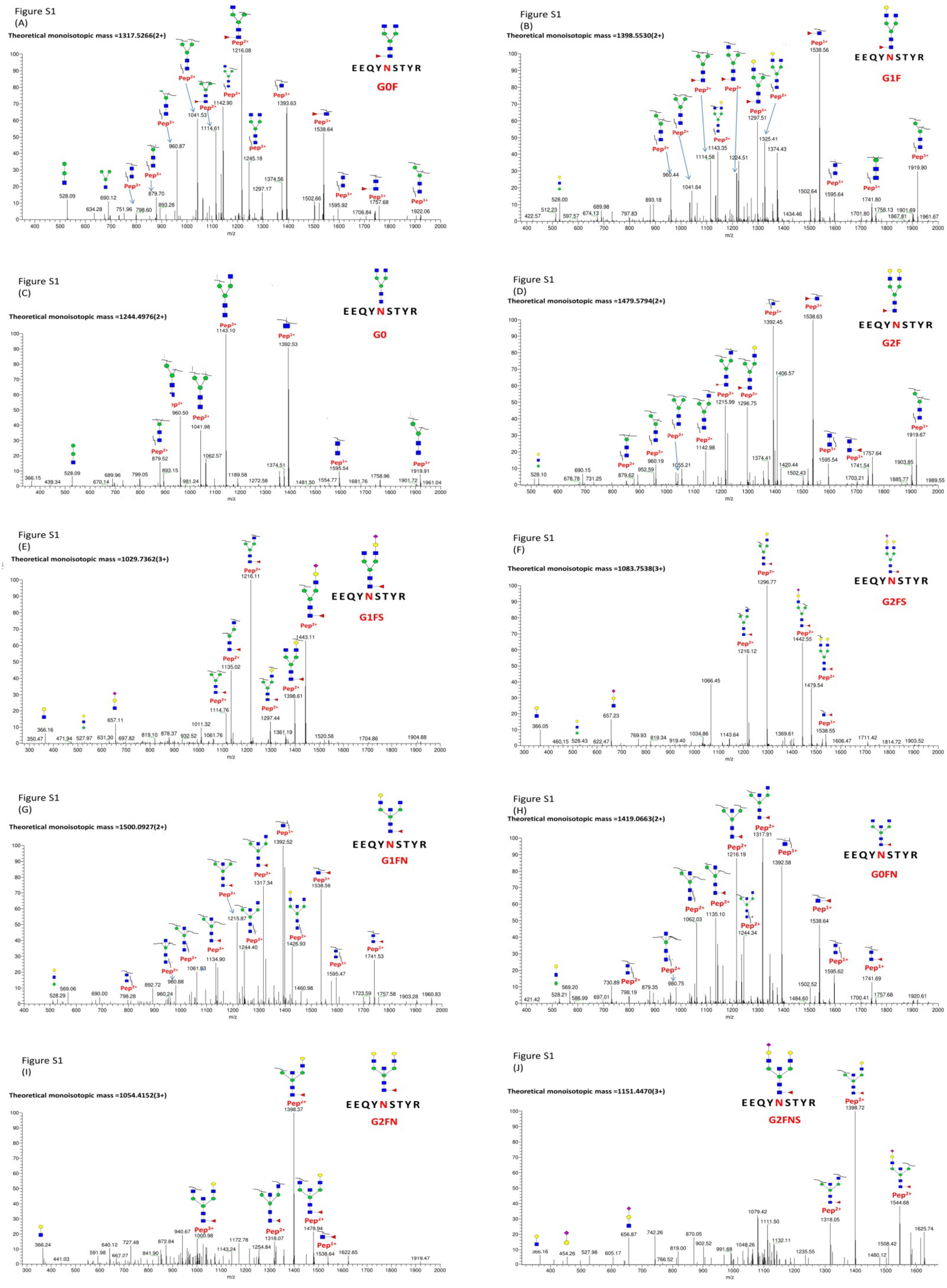

Supplementary Figure 1. MS^2 spectrum of 10 serum IgG-$_1$ glycoforms. Each glycan structure was confirmed using the fragment ions generated by the collision-induced dissociation.

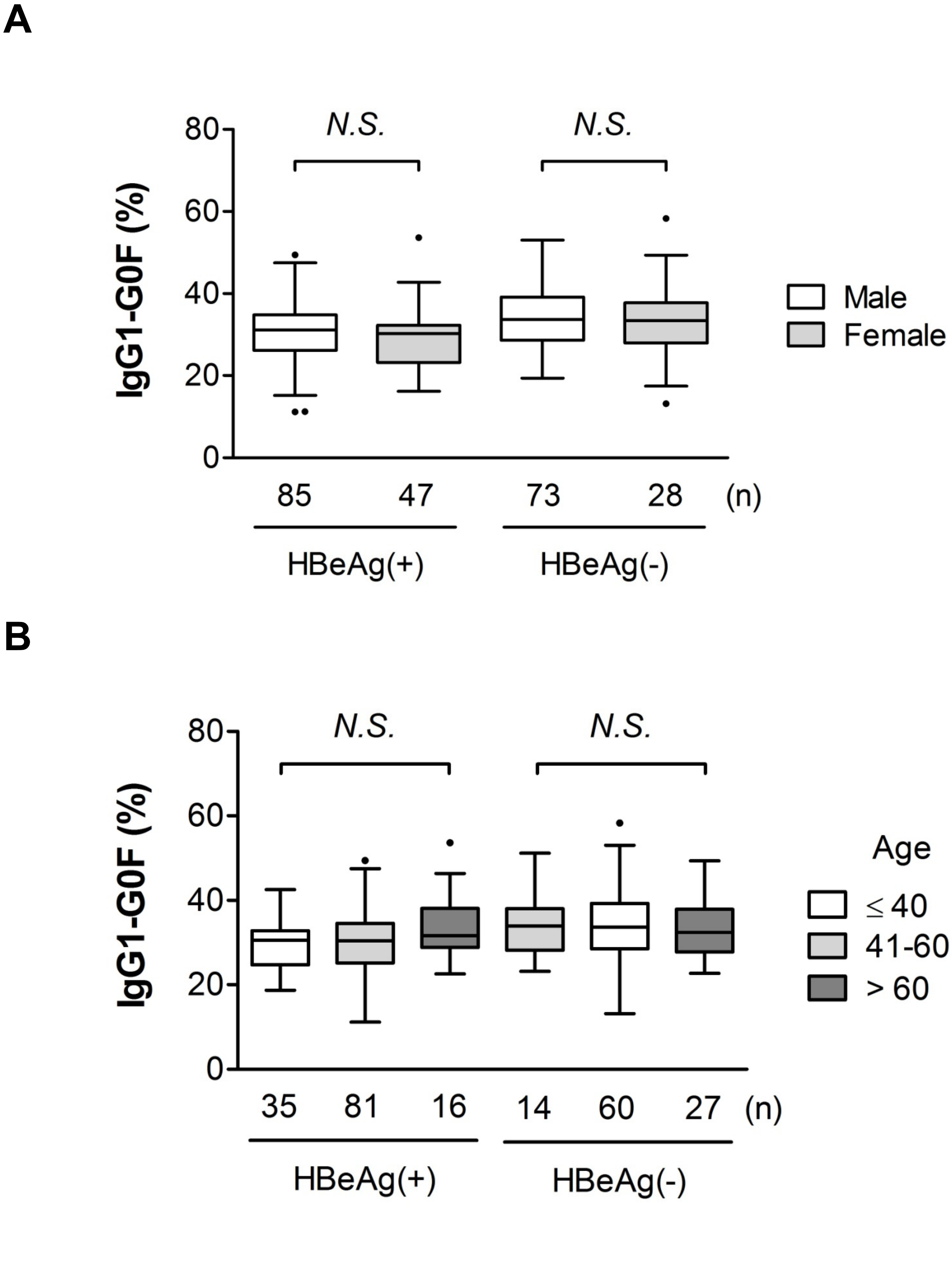

Supplementary Figure 2. Serum IgG1-G0F level in patients with CHB was not associated with age or sex. Comparisons of baseline level of IgG1-G0F (A) between male and female or (B) in different ages, are shown as Tukey's box-and-whisker plots. *P*-values in (A) and (B) are from Mann–Whitney *U* tests and Kruskal-Wallis tests, respectively. HBeAg, hepatitis B virus e antigen.

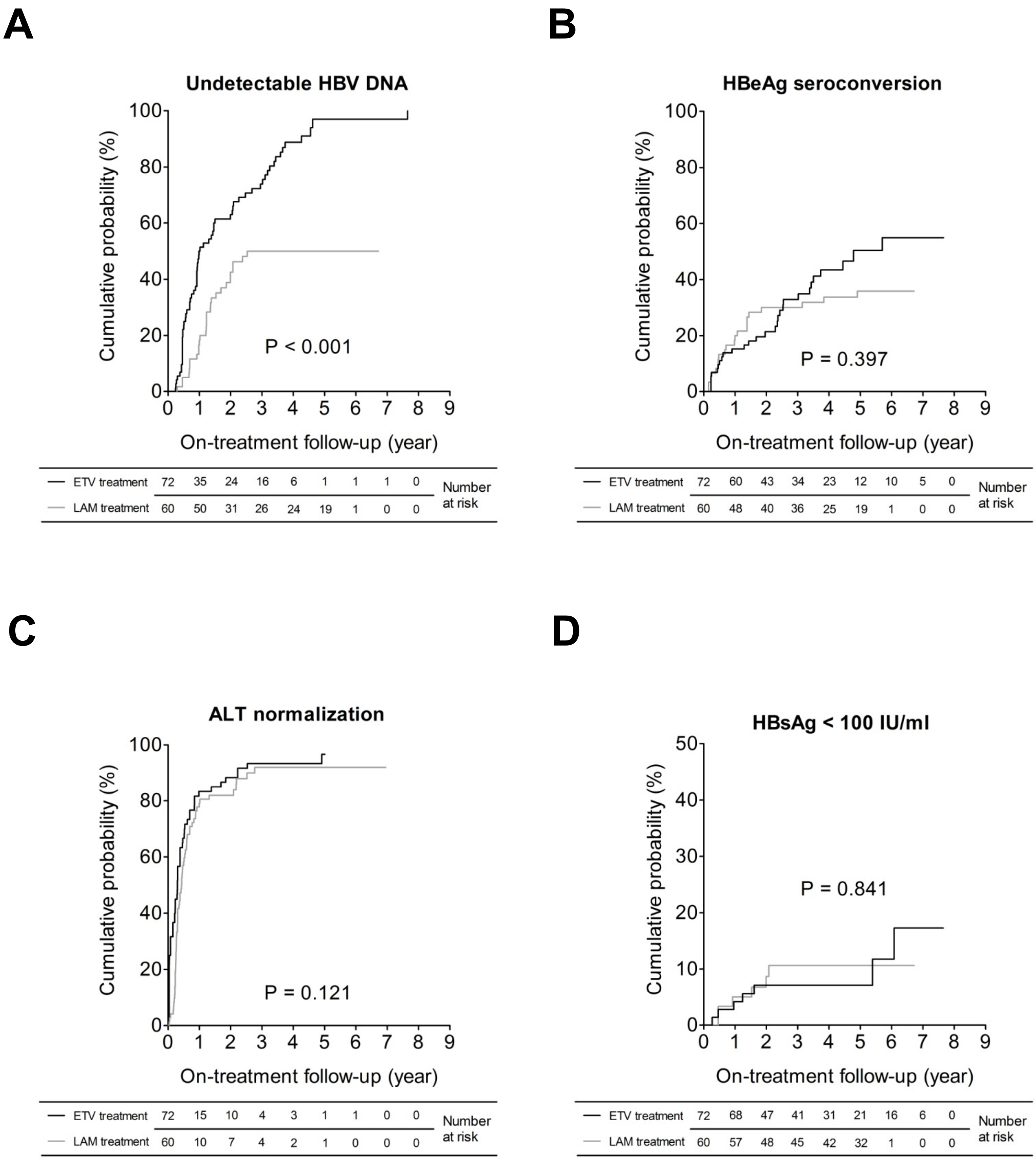

Supplementary Figure 3. Comparisons of long-term treatment efficacies between entecavir and lamivudine in HBeAg-positive CHB. Kaplan-Meier analyses of efficacies of ETV (black line) and LAM (gray line) on (A) virological response, (B) HBeAg seroconversion, (C) ALT normalization, and (D) HBsAg <100 IU/ml, are shown. *P*-values are obtained from log-rank tests. ALT, alanine aminotransferase; ETV, entecavir; HBeAg, hepatitis B virus e antigen; HBsAg, hepatitis B virus surface antigen; HBV, hepatitis B virus; LAM, lamivudine.

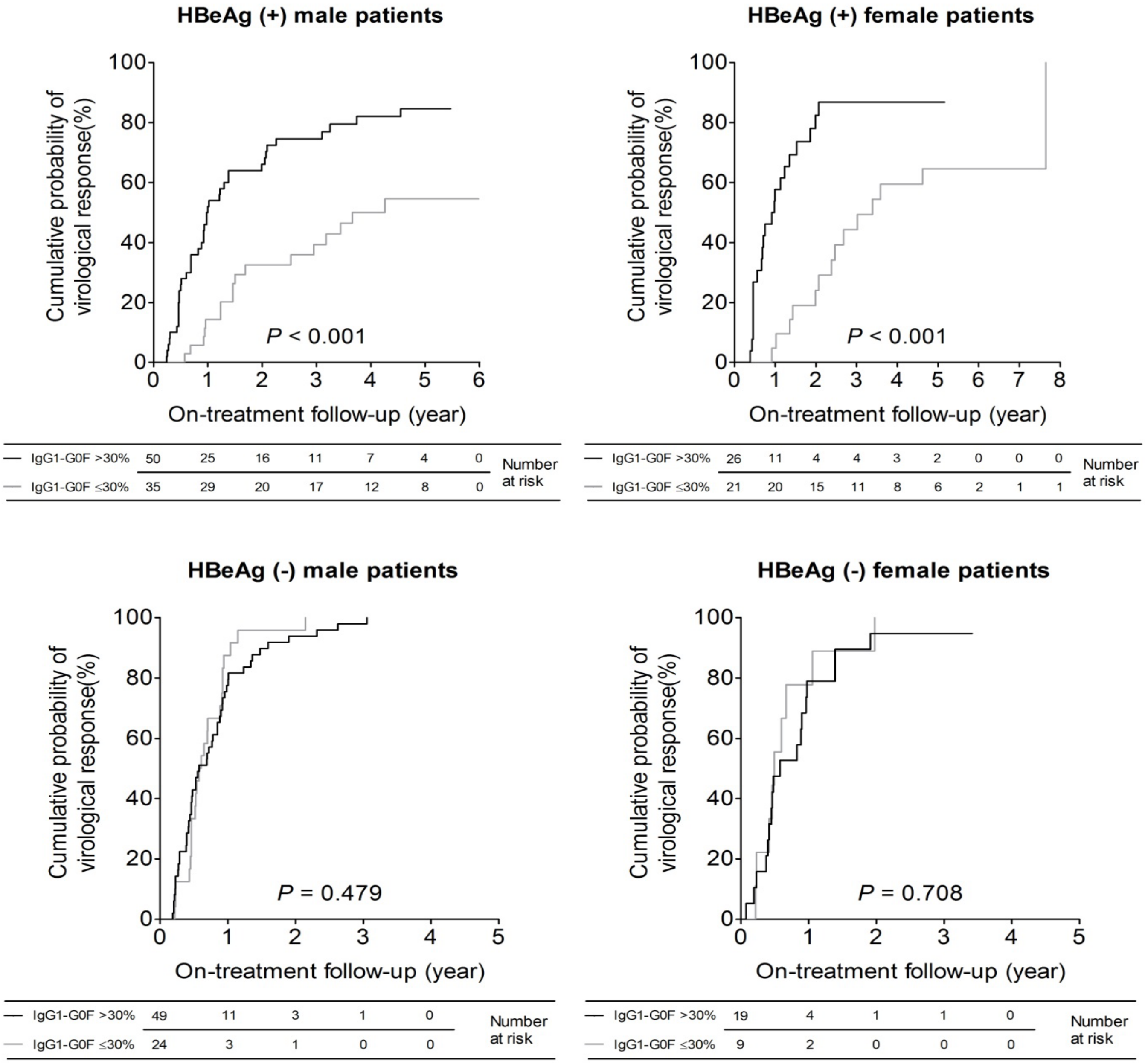

Supplementary Figure 4. Comparisons of the discrimination power of baseline IgG1-G0F on virological response between male and female patients. Kaplan-Meier analyses of virological response in male or female patients with chronic HBV infection are shown. *P*-values are obtained from log-rank tests. HBeAg, hepatitis B virus e antigen.

A

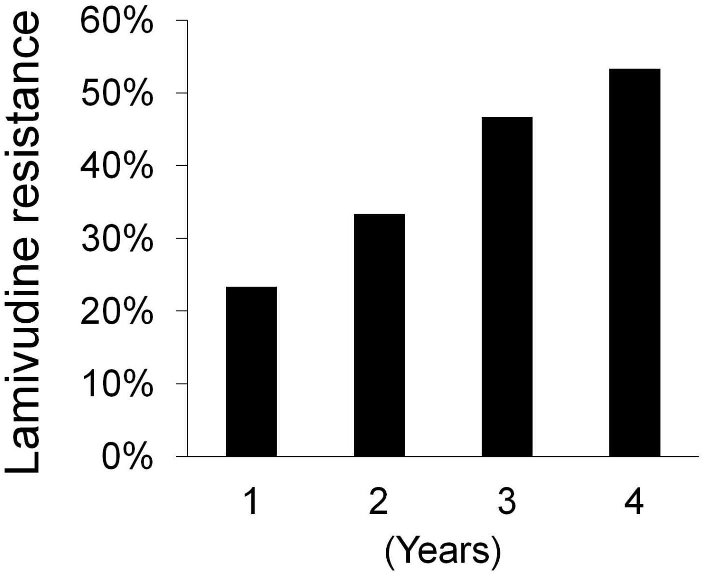

B

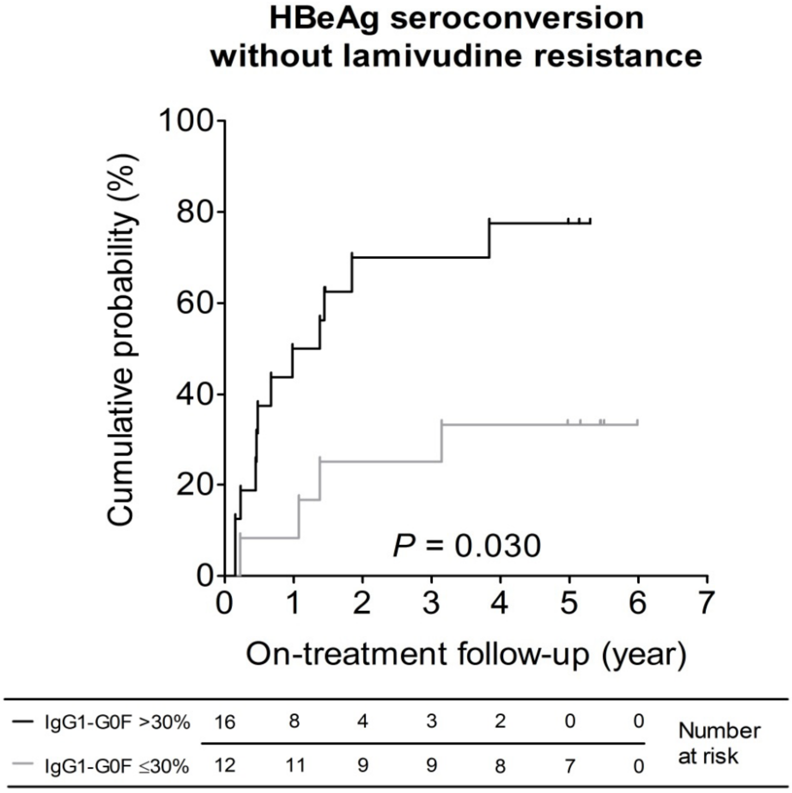

Supplementary Figure 5. An increase in HBeAg seroconversion by a high baseline level of IgG1-G0F in non-lamivudine-resistant HBeAg-positive patients. (A) Accumulative frequency of lamivudine resistance in HBeAg-positive patients (n = 60) at year1 to 4 is shown. (B) A Kaplan-Meier analysis of HBeAg seroconversion in lamivudine resistance-free patients with or without baseline IgG1-G0F >30% is shown. *P*-value is obtained from log-rank test.
